# Supplementary material for: How development and survival combine to determine the thermal sensitivity of insects
Source: PLoS One. 2024 Jan 30;19(1):e0291393. doi: 10.1371/journal.pone.0291393 (PMC10826953; doi:10.1371/journal.pone.0291393)
Supplement: S1 File — (DOCX) [file pone.0291393.s001.docx]

**Supplement 1**: List of species and location of populations included in analyses


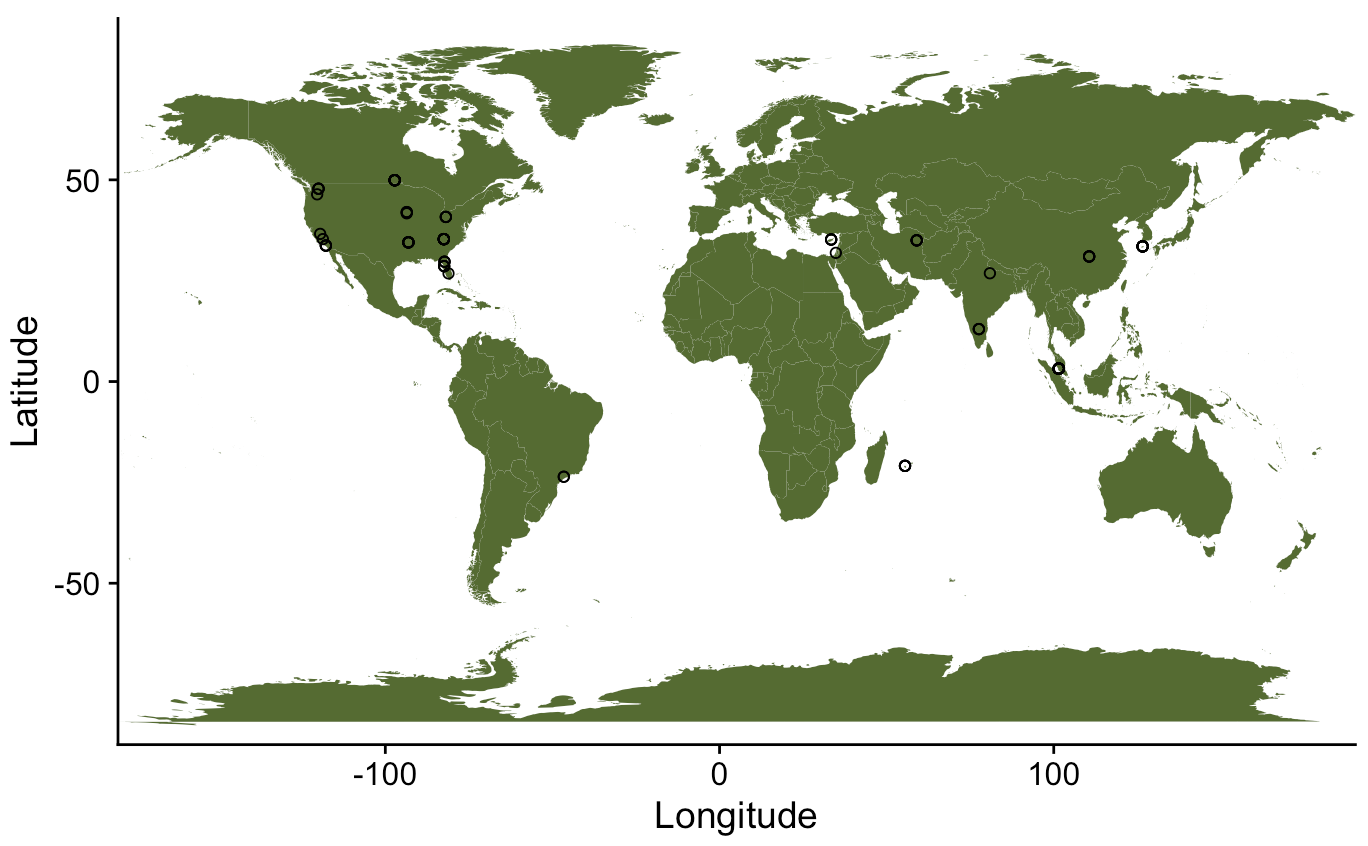


Figure S1. Location of study populations in the analytical data set. Figure generated using the R package “maps”^1^.

Table S1. List of 26 species constituting the analytical data set.

| Species | family |
| --- | --- |
| *Herpetogramma phaeopteralis* | Crambidae |
| *Chilo sacchariphagus* | Crambidae |
| *Keiferia lycopersicella* | Gelechiidae |
| *Ascotis selenaria* | Geometridae |
| *Marmara gulosa* | Gracillariidae |
| *Spalgis epius* | Lycaenidae |
| *Mamestra configurata* | Noctuidae |
| *Peridroma saucia* | Noctuidae |
| *Chilo auricilius* | Noctuidae |
| *Lacanobia subjuncta* | Noctuidae |
| *Spodoptera exigua* | Noctuidae |
| *Cerconota anonella* | Oecophoridae |
| *Ectomyelois ceratoniae* | Phycitidae |
| *Ephestia figulilella* | Phycitidae |
| *Homadaula anisocentra* | Plutellidae |
| *Metisa plana* | Psychidae |
| *Pteroma pendula* | Psychidae |
| *Elasmopalpus lignosellus* | Pyralidae |
| *Ameyelois transitella* | Pyralidae |
| *Orthopygia glaucinalis* | Pyralidae |
| *Lista haraldusalis* | Pyralidae |
| *Rhyacionia frustrana* | Tortricidae |
| *Ancylis comptana* | Tortricidae |
| *Episimus utilis* | Tortricidae |
| *Argyrotaenia velutinana* | Tortricidae |
| *Cydia pomonella* | Tortricidae |


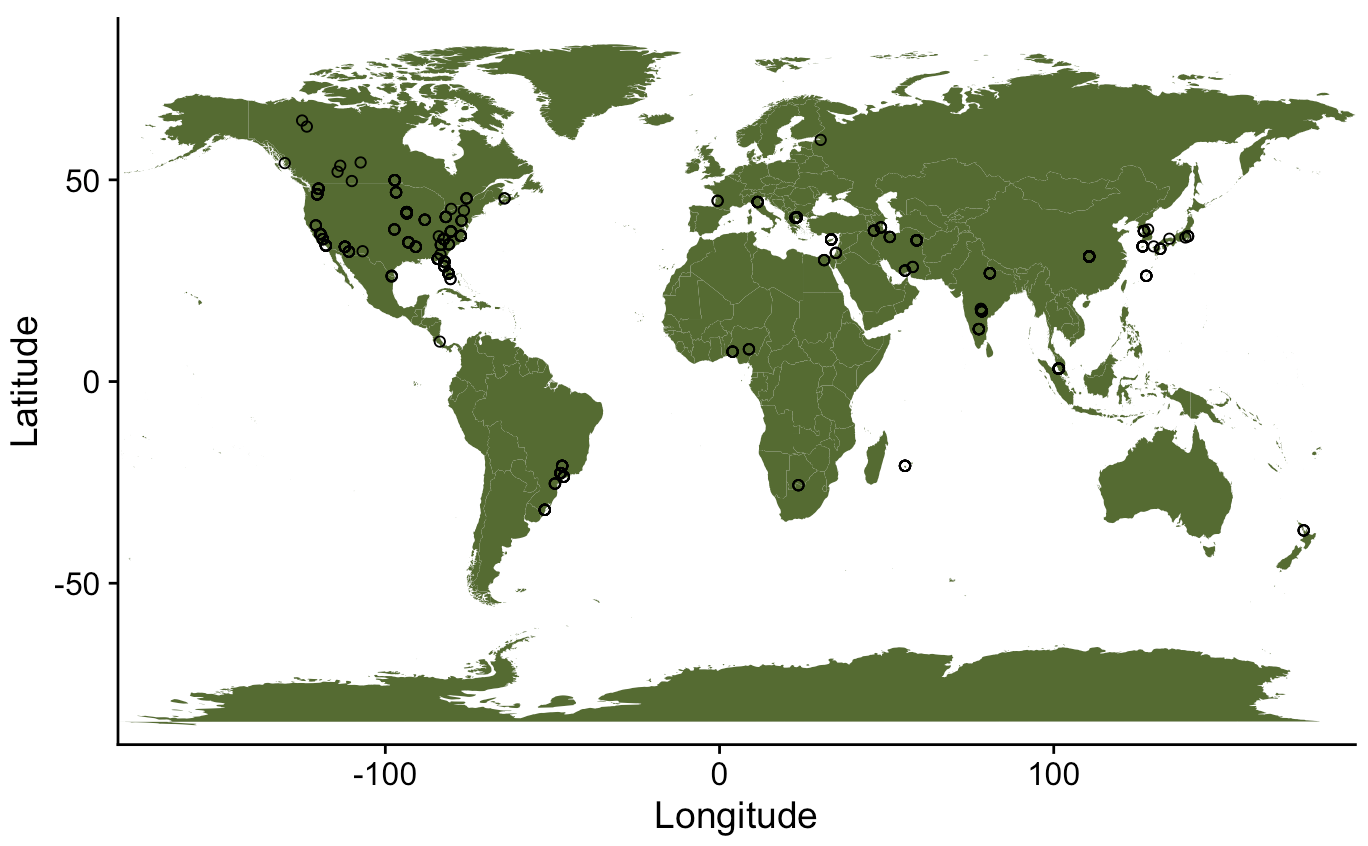


**Figure S2**. Location of study populations included in the full data set (analysis of this data set is reported in supplement 2). Figure generated using the R package “maps”^1^.

Table S2. List of 89 species constituting the full data set.

| Species | Family |
| --- | --- |
| *Utethesia ornatrix* | Arctiidae |
| *Bedellia somnulentella* | Bellelliidae |
| *Carposina sasakii* | Carposinidae |
| *Cochylis hospes* | Cochylidae |
| *Herpetogramma phaeopteralis* | Crambidae |
| *Chilo sacchariphagus* | Crambidae |
| *Pediasia trisecta* | Crambidae |
| *Diaphania nitidalis* | Crambidae |
| *Diaphania indica* | Crambidae |
| *Ostrinia nubilalis* | Crambidae |
| *Diatraea grandiosella* | Crambidae |
| *Palpita nigropunctalis* | Crambidae |
| *Neoleucinodes elegantalis* | Crambidae |
| *Cnaphalocrocis medinalis* | Crambidae |
| *Stenoma catenifer* | Elachistidae |
| *Lymantria albescens* | Erebidae |
| *Lymantria xylina* | Erebidae |
| *Keiferia lycopersicella* | Gelechiidae |
| *Anarsia lineatella* | Gelechiidae |
| *Tuta absoluta* | Gelechiidae |
| *Pectinophora gossypiella* | Gelechiidae |
| *Aproaerema modicella* | Gelechiidae |
| *Phthorimaea operculella* | Gelechiidae |
| *Ascotis selenaria* | Geometridae |
| *Marmara gulosa* | Gracillariidae |
| *Phyllonorycter blancardella* | Gracillariidae |
| *Spalgis epius* | Lycaenidae |
| *Lycaeides argyrognomom* | Lycaenidae |
| *Mamestra configurata* | Noctuidae |
| *Peridroma saucia* | Noctuidae |
| *Argyrogramma albostriata* | Noctuidae |
| *Chilo auricilius* | Noctuidae |
| *Sesamia nonagrioides* | Noctuidae |
| *Spodoptera cosmioides* | Noctuidae |
| *Lacanobia subjuncta* | Noctuidae |
| *Spodoptera exigua* | Noctuidae |
| *Panolis flammea* | Noctuidae |
| *Heliothis subflexa* | Noctuidae |
| *Heliothis virescens* | Noctuidae |
| *Heliothis zea* | Noctuidae |
| *Hypantria cunea* | Noctuidae |
| *Leucania separata* | Noctuidae |
| *Pseudaletia unipuncta* | Noctuidae |
| *Simyra henrici* | Noctuidae |
| *Trichoplusia ni* | Noctuidae |
| *Egira curialis* | Noctuidae |
| *Spodoptera litura* | Noctuidae |
| *Naranga aenescens* | Noctuidae |
| *Sesamia cretica* | Noctuidae |
| *Anticarsia gemmatalis* | Noctuidae |
| *Inachis io* | Nymphalidae |
| *Danaus plexippus* | Nymphalidae |
| *Cerconota anonella* | Oecophoridae |
| *Atrophaneura alcinous* | Papilionidae |
| *Sericinus montela* | Papilionidae |
| *Ectomyelois ceratoniae* | Phycitidae |
| *Ephestia calidella* | Phycitidae |
| *Ephestia figulilella* | Phycitidae |
| *Homadaula anisocentra* | Plutellidae |
| *Plutella xylostella* | Plutellidae |
| *Metisa plana* | Psychidae |
| *Pteroma pendula* | Psychidae |
| *Diatraea saccharalis* | Pyralidae |
| *Dioryctria amatella* | Pyralidae |
| *Ameyelois transitella* | Pyralidae |
| *Cactoblastis cactorum* | Pyralidae |
| *Maruca vitrata* | Pyralidae |
| *Euzopherodes vapidella* | Pyralidae |
| *Elasmopalpus lignosellus* | Pyralidae |
| *Orthopygia glaucinalis* | Pyralidae |
| *Hypocosmia pyrochroma* | Pyralidae |
| *Lista haraldusalis* | Pyralidae |
| *Cadra cautella* | Pyralidae |
| *Corcyra cephalonica* | Pyralidae |
| *Plodia interpunctella* | Pyralidae |
| *Diatraea lineolata* | Pyralidae |
| *Hypsipyla grandella* | Pyralidae |
| *Attacus ricini* | Saturnidae |
| *Platynota idaeusalis* | Tortricidae |
| *Rhyacionia frustrana* | Tortricidae |
| *Ancylis comptana* | Tortricidae |
| *Cydia pomonella* | Tortricidae |
| *Episimus utilis* | Tortricidae |
| *Ctenopseustis obliquana* | Tortricidae |
| *Adoxophyes orana* | Tortricidae |
| *Choristoneura fumiferana* | Tortricidae |
| *Argyrotaenia velutinana* | Tortricidae |
| *Croesia curvalana* | Tortricidae |
| *Lobesia botrana* | Tortricidae |

References

1. Becker OScbRA, Minka ARWRvbRBEbTP, Deckmyn. A (2022). _maps: Draw Geographical Maps_. R package  version 3.4.1, <https://CRAN.R-project.org/package=maps>.
