# Supplementary material for: How development and survival combine to determine the thermal sensitivity of insects
Source: PLoS One. 2024 Jan 30;19(1):e0291393. doi: 10.1371/journal.pone.0291393 (PMC10826953; doi:10.1371/journal.pone.0291393)
Supplement: S2 File — (DOCX) [file pone.0291393.s002.docx]

**Supplement 2:** Model results from the “analytical” and “full” data sets

We performed the analyses described in the “analyses” section on two data sets the “Analytical data set”, which includes only sets from which all three performance curves could be estimated and the full data set, which also includes studies reporting at least one of them. The **“full dataset”** consisted of overlapping suites of sets reporting development time (n = 75 species, 173 sets, 24% inferred locations) and/or survival (n = 54 species, 117 sets, 31% inferred locations) data.

**Table 1:** Model results from the “analytical dataset”. Interactive or additive models were chosen by AIC. When the models did not differ significantly, the additive model was chosen. Significant terms are marked with asterisks (*: p < 0.05, **: p < 0.01, ***: p < 0.001)

| 1. **D_h_** |  |  |  |
| --- | --- | --- | --- |
| **Fixed effects** | **Estimate** | **SE** | **t-value** |
| absolute latitude | -0.06 | 0.04 | -1.32 |
| egg | 36.20 | 1.39 | 26.09 |
| larva | 36.11 | 1.40 | 25.69 |
| pupa | 36.85 | 1.39 | 26.47 |
| larva:absolute latitude | -0.01 | 0.03 | -0.34 |
| pupa:absolute latitude | -0.06 | 0.03 | -2.12 |
| **Random effects** | **SD** |  |  |
| species | 2.12 |  |  |
| residual | 0.82 |  |  |
| N = 35 obs., 20 species |  |  |  |
| 1. **D_l_** |  |  |  |
| **Fixed effects** | **Estimate** | **SE** | **t-value** |
| absolute latitude | -0.15 | 0.04 | -3.90** |
| egg | 25.68 | 1.28 | 20.01 |
| larva | 25.37 | 1.32 | 19.20 |
| pupa | 26.45 | 1.33 | 19.88 |
| **Random effects** | **SD** |  |  |
| Species | 1.91 |  |  |
| residual | 1.28 |  |  |
| N = 37 obs., 21 species |  |  |  |
| 1. **D_breadth_** |  |  |  |
| **Fixed effects** | **Estimate** | **SE** | **t-value** |
| absolute latitude | 0.07 | 0.02 | 2.89* |
| egg | 11.16 | 0.86 | 12.97 |
| larva | 11.29 | 0.90 | 12.53 |
| pupa | 9.54 | 0.86 | 11.09** |
| **Random effects** | **SD** |  |  |
| species | 1.16 |  |  |
| residual | 0.90 |  |  |
| N = 26 obs., 15 species |  |  |  |
| 1. **S_h_** |  |  |  |
| **Fixed effects** | **Estimate** | **SE** | **t-value** |
| absolute latitude | -0.05 | 0.05 | -0.98 |
| egg | 35.32 | 1.68 | 21.02 |
| larva | 35.32 | 1.70 | 20.82 |
| pupa | 36.43 | 1.68 | 21.63 |
| larva:absolute latitude | -0.05 | 0.03 | -1.69 |
| pupa:absolute latitude | -0.08 | 0.03 | -2.92* |
| **Random effects** | **SD** |  |  |
| species | 2.64 |  |  |
| residual | 0.85 |  |  |
| N = 35 obs., 20 species |  |  |  |
| 1. **S_l_** |  |  |  |
| **Fixed effects** | **Estimate** | **SE** | **t-value** |
| absolute latitude | -0.20 | 0.06 | -3.46** |
| egg | 22.29 | 1.90 | 11.70 |
| larva | 22.23 | 1.96 | 11.34 |
| pupa | 21.38 | 1.97 | 10.83 |
| **Random effects** | **SD** |  |  |
| species | 2.83 |  |  |
| residual | 1.91 |  |  |
| N = 37 obs., 21 species |  |  |  |
| 1. **S_breadth_** |  |  |  |
| **Fixed effects** | **Estimate** | **SE** | **t-value** |
| absolute latitude | 0.20 | 0.08 | 2.67* |
| egg | 11.70 | 2.43 | 4.82 |
| larva | 12.09 | 2.49 | 4.85 |
| pupa | 16.87 | 2.47 | 6.84** |
| larva:absolute latitude | -0.08 | 0.05 | -1.60 |
| pupa:absolute latitude | -0.17 | 0.05 | -3.36** |
| **Random effects** | **SD** |  |  |
| species | 3.51 |  |  |
| residual | 1.52 |  |  |
| N = 26 obs., 15 species |  |  |  |
| 1. **P_h_** |  |  |  |
| **Fixed effects** | **Estimate** | **SE** | **t-value** |
| absolute latitude | -0.06 | 0.04 | -1.36 |
| egg | 35.91 | 1.48 | 24.33 |
| larva | 36.42 | 1.49 | 24.50 |
| pupa | 36.87 | 1.48 | 24.93 |
| larva:absolute latitude | -0.05 | 0.02 | -2.31* |
| pupa:absolute latitude | -0.08 | 0.02 | -3.66** |
| **Random effects** | **SD** |  |  |
| species | 2.37 |  |  |
| residual | 0.64 |  |  |
| N = 35 obs., 20 species |  |  |  |
| 1. **P_l_** |  |  |  |
| **Fixed effects** | **Estimate** | **SE** | **t-value** |
| absolute latitude | -0.14 | 0.04 | -3.70** |
| egg | 25.41 | 1.25 | 20.26 |
| larva | 24.99 | 1.32 | 18.97 |
| pupa | 26.28 | 1.33 | 19.74 |
| **Random effects** | **SD** |  |  |
| species | 1.55 |  |  |
| residual | 1.89 |  |  |
| N = 37 obs., 21 species |  |  |  |
| 1. **P_breadth_** |  |  |  |
| **Fixed effects** | **Estimate** | **SE** | **t-value** |
| absolute latitude | 0.03 | 0.04 | 0.68 |
| egg | 11.58 | 1.33 | 8.70 |
| larva | 12.09 | 1.40 | 8.62 |
| pupa | 10.14 | 1.33 | 7.61 |
| **Random effects** | **SD** |  |  |
| species | 1.73 |  |  |
| residual | 1.54 |  |  |
| N = 26 obs., 15 species |  |  |  |

**Table 2:** Model results from the full datasets. Interactive or additive models were chosen by AIC. When the models did not differ significantly, the additive model was chosen.

| 1. **D_h_** |  |  |  |
| --- | --- | --- | --- |
| **Fixed effects** | **Estimate** | **SE** | **t-value** |
| absolute latitude | -0.05 | 0.03 | -1.62 |
| egg | 35.32 | 1.02 | 34.56 |
| larva | 34.97 | 1.01 | 34.66 |
| pupa | 34.91 | 1.01 | 34.52 |
| **Random effects** | **SD** |  |  |
| species | 2.60 |  |  |
| residual | 1.07 |  |  |
| N = 84 obs., 41 species |  |  |  |
| 1. **D_l_** |  |  |  |
| **Fixed effects** | **Estimate** | **SE** | **t-value** |
| absolute latitude | -0.96 | 0.021 | -4.64 |
| egg | 23.74 | 0.72 | 33.1 |
| larva | 23.45 | 0.73 | 32.22 |
| pupa | 24.17 | 0.73 | 33.27 |
| **Random effects** | **SD** |  |  |
| species | 1.81 |  |  |
| residual | 1.18 |  |  |
| N = 167 obs., 71 species |  |  |  |
| 1. **D_breadth_** |  |  |  |
| **Fixed effects** | **Estimate** | **SE** | **t-value** |
| absolute latitude | 0.031 | 0.02 | 1.28 |
| egg | 12.47 | 0.86 | 14.51 |
| larva | 12.01 | 0.84 | 14.39 |
| pupa | 11.09 | 0.84 | 13.14 |
| **Random effects** | **SD** |  |  |
| species | 3.06 |  |  |
| residual | 1.50 |  |  |
| N = 82 obs., 39 species |  |  |  |
| 1. **S_h_** |  |  |  |
| **Fixed effects** | **Estimate** | **SE** | **t-value** |
| absolute latitude | -0.09 | 0.034 | -2.50 |
| egg | 34.89 | 1.32 | 26.44 |
| larva | 34.68 | 1.36 | 25.45 |
| pupa | 34.64 | 1.34 | 25.79 |
| **Random effects** | **SD** |  |  |
| species | 2.35 |  |  |
| residual | 1.90 |  |  |
| N = 65obs., 36 species |  |  |  |
| 1. **S_l_** |  |  |  |
| **Fixed effects** | **Estimate** | **SE** | **t-value** |
| absolute latitude | -0.26 | 0.49 | -5.37 |
| egg | 23.51 | 1.60 | 14.65 |
| larva | 20.89 | 1.49 | 13.99 |
| pupa | 19.28 | 1.81 | 10.66 |
| absolute latitude: larva | 0.12 | 0.05 | 2.68 |
| absolute latitude: pupa | 0.14 | 0.05 | 2.6 |
| **Random effects** | **SD** |  |  |
| species | 2.80 |  |  |
| residual | 1.75 |  |  |
| N = 61 obs., 34 species |  |  |  |
| 1. **S_breadth_** |  |  |  |
| **Fixed effects** | **Estimate** | **SE** | **t-value** |
| absolute latitude | 0.11 | 0.05 | 2.30 |
| egg | 14.32 | 1.83 | 7.83 |
| larva | 11.36 | 1.88 | 6.05 |
| pupa | 13.83 | 1.91 | 7.24 |
| **Random effects** | **SD** |  |  |
| species | 2.40 |  |  |
| residual | 3.28 |  |  |
| N = 41 obs., 24 species |  |  |  |
| 1. **P_h_** |  |  |  |
| **Fixed effects** | **Estimate** | **SE** | **t-value** |
| absolute latitude | -0.07 | 0.04 | -1.95 |
| egg | 35.00 | 1.25 | 28.01 |
| larva | 34.86 | 1.27 | 27.36 |
| pupa | 34.65 | 1.27 | 27.25 |
| **Random effects** | **SD** |  |  |
| species | 2.32 |  |  |
| residual | 1.08 |  |  |
| N = 62 obs., 36 species |  |  |  |
| 1. **P_l_** |  |  |  |
| **Fixed effects** | **Estimate** | **SE** | **t-value** |
| absolute latitude | -0.12 | 0.03 | -4.31 |
| egg | 24.61 | 0.95 | 25.82 |
| larva | 24.69 | 1.00 | 24.80 |
| pupa | 25.11 | 0.97 | 25.80 |
| **Random effects** | **SD** |  |  |
| species | 1.60 |  |  |
| residual | 1.80 |  |  |
| N = 96 obs., 46 species |  |  |  |
| 1. **P_breadth_** |  |  |  |
| **Fixed effects** | **Estimate** | **SE** | **t-value** |
| absolute latitude | 0.02 | 0.04 | 0.44 |
| egg | 11.18 | 1.26 | 8.86 |
| larva | 11.50 | 1.32 | 8.74 |
| pupa | 10.77 | 1.30 | 8.31 |
| **Random effects** | **SD** |  |  |
| species | 2.095 |  |  |
| residual | 1.59 |  |  |
| N = 60 obs., 34 species |  |  |  |
|  |  |  |  |
|  |  |  |  |
