# Supplementary material for: How development and survival combine to determine the thermal sensitivity of insects
Source: PLoS One. 2024 Jan 30;19(1):e0291393. doi: 10.1371/journal.pone.0291393 (PMC10826953; doi:10.1371/journal.pone.0291393)
Supplement: S3 File — (DOCX) [file pone.0291393.s003.docx]

**Supplement 3**: Phylogenetic analyses

We searched for 11 full-length, commonly sequenced (Wahlberg and Wheat, 2008) genomic DNA markers (Table 1), across all 102 species using GeneDumper, a toolkit designed to search GenBank for certain loci given a taxonomy. The taxonomy used for input was generated from the NCBI taxonomy database in R v3.6.3 using the *tax_name* command from the package taxise. Sequences were validated and curated using a detailed pipeline available in the GeneDumper toolkit to remove paralogs, uninformative and mislabeled sequences. Five species were not available on Genbank, and in those cases, we opted to search for and utilize a congener as a surrogate (Table 2). Given that sampling covered a significant breadth of Lepidoptera, utilizing surrogates within the same genus will have a negligible effect on downsteam use of the tree in phylogenetic linear mixed modeling.

Sequences were aligned with mafft v7.294b (Katoh & Standley, 2014) employing the --adjustdirection and --localpair options. AliView v1.26 (Larsson, 2014) was used to view and edit the alignments by hand if needed. Nearly identical sequences (>95% identity and within 50 bp in length) with the same species label were randomly chosen. Sequences covering different areas of the same loci (<100 bp overlap) within the same species were merged together using a degenerate consensus technique employed in AliView. Locus alignments containing 10 or less species were removed, leaving a total of 8 usable loci. A supermatrix for the final dataset was generated using FasConCat-G v1.0 (Kück & Longo, 2014). The supermatrix contained 64% missing data, but all 102 species were represented by the common barcode *COI*, followed by a *Ef-1a* representation (50% coverage) and *Wgl* and *CAD* (each with 32% coverage).

An unpartitioned maximum likelihood tree was built using RAxML-NG v0.9.0 (Kozlov et al. 2019) under a GTR-G substitution model. The phylogeny was constrained by superfamily, due to the small number of species across a large order that diverged over 100 million years ago (Kawahara et al. 2019). We ran 60 analyses (30 starting from random trees and 30 starting from a maximum parsimony tree). The phylogeny with a log-likelihood closest to 0 was chosen as the final phylogeny (-119586.886). FigTree v2.0 (Rambaut, 2010) was used to visualize and root the tree to superfamily Tineoidea (Mitter et al. 2017, Kawahara et al. 2019, Kristensen et al. 2007 ). See Figure 1 for final phylogeny colored by superfamily.

All analyses were run using the high-performance cluster (HPC) at the University of Florida, HiperGator 2.0. Raw data and cleaned products (uncleaned FASTA files, final alignment and final tree file) are available (DOI: 10.5061/dryad.qjq2bvqk4 ).

Table 1: The names and lengths of the 11 loci used as the starting sequence for input into GeneDumper.

| Locus Name | Locus Length (bp) |
| --- | --- |
| *ArgKin* | 596 |
| *CAD* | 2,211 |
| *COI_trnL_COII* | 2,271 (COI: 1,531; trnL: 67; COII: 673) |
| *DDC* | 957 |
| *EF1a* | 1,240 |
| *GAPDH* | 609 |
| *IDH* | 709 |
| *MDH* | 733 |
| *RpS2* | 474 |
| *RpS5* | 614 |
| *Wgl* | 453 |

Table 2: Species substitutions made due to lack of DNA information.

| Species | Replacement Species |
| --- | --- |
| *Ephestia calidella* | *Ephestia columbiella* |
| *Episimus utilis* | *Episimus tyrius* |
| *Euzopherodes vapidella* | *Euzopherodes allocrossa* |
| *Marmara gulosa* | *Marmara arbutiella* |
| *Naranga aenescens* | *Protodeltote albidula* |

Figure 1: Final phylogram colored by superfamily, rooted to Tineoidea.


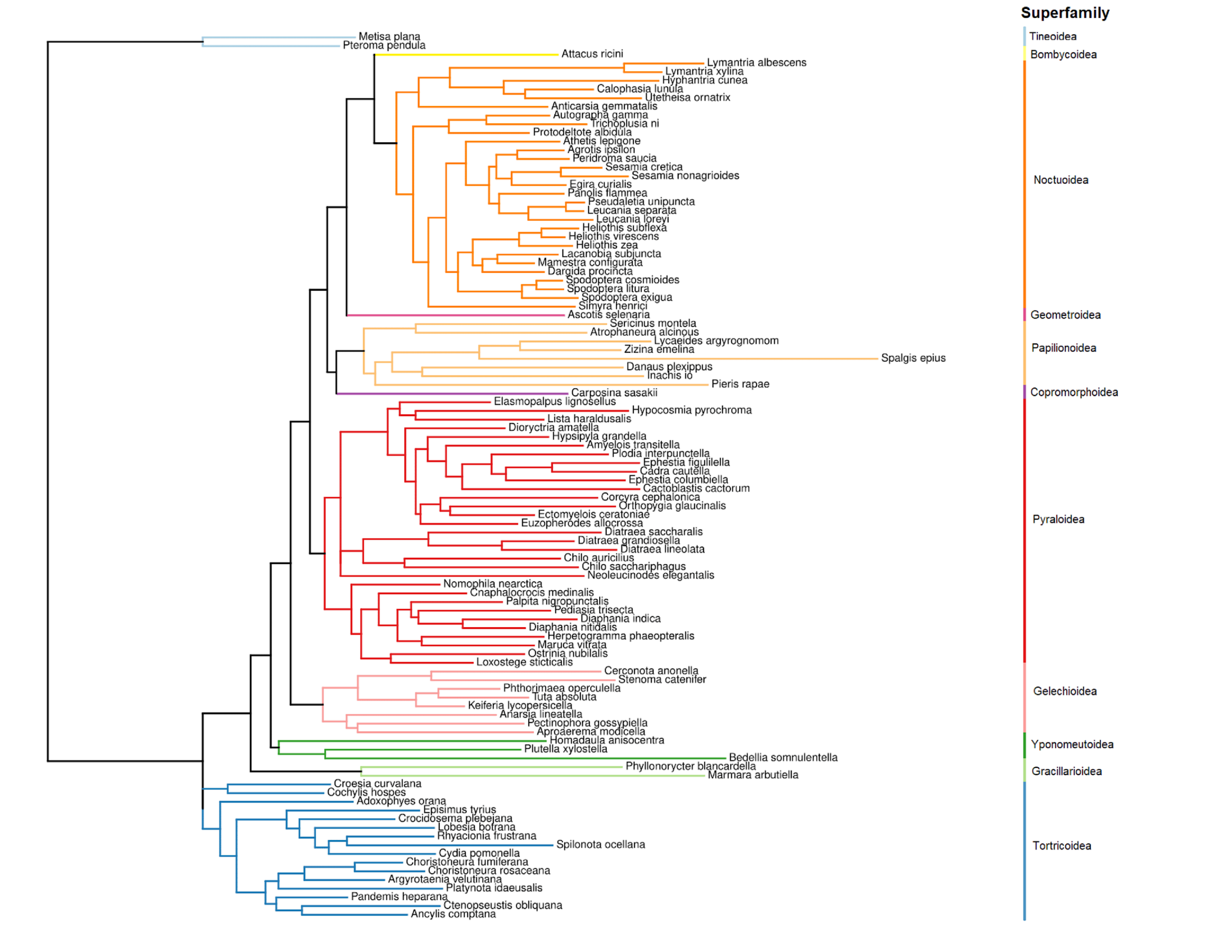


References

Katoh, K. and Standley, D.M., 2014. MAFFT: iterative refinement and additional methods. Multiple sequence alignment methods, pp.131-146.

Kawahara, A.Y., Plotkin, D., Espeland, M., Meusemann, K., Toussaint, E.F., Donath, A., Gimnich, F., Frandsen, P.B., Zwick, A., Dos Reis, M. and Barber, J.R., 2019. Phylogenomics reveals the evolutionary timing and pattern of butterflies and moths. *Proceedings of the National Academy of Sciences*, *116*(45), pp.22657-22663.

Kristensen, N.P., Scoble, M.J. and Karsholt, O.L.E., 2007. Lepidoptera phylogeny and systematics: the state of inventorying moth and butterfly diversity. *Zootaxa*, *1668*(1), pp.699-747.

Kück, P. and Longo, G.C., 2014. FASconCAT-G: extensive functions for multiple sequence alignment preparations concerning phylogenetic studies. *Frontiers in zoology*, *11*, pp.1-8.

Kozlov, A.M., Darriba, D., Flouri, T., Morel, B. and Stamatakis, A., 2019. RAxML-NG: a fast, scalable and user-friendly tool for maximum likelihood phylogenetic inference. *Bioinformatics*, *35*(21), pp.4453-4455.

Larsson, A., 2014. AliView: a fast and lightweight alignment viewer and editor for large datasets. *Bioinformatics*, *30*(22), pp.3276-3278.

Mitter, C., Davis, D.R. and Cummings, M.P., 2017. Phylogeny and evolution of Lepidoptera. *Annual Review of Entomology*, *62*, pp.265-283.

Wahlberg, N. and Wheat, C.W., 2008. Genomic outposts serve the phylogenomic pioneers: designing novel nuclear markers for genomic DNA extractions of Lepidoptera. *Systematic biology*, *57*(2), pp.231-242.
