# Supplementary material for: How development and survival combine to determine the thermal sensitivity of insects
Source: PLoS One. 2024 Jan 30;19(1):e0291393. doi: 10.1371/journal.pone.0291393 (PMC10826953; doi:10.1371/journal.pone.0291393)
Supplement: S4 File — (DOCX) [file pone.0291393.s004.docx]

**Supplement 4:** Models with phylogenetic correction

1. Phylogenetic tree used in analysis of development variables


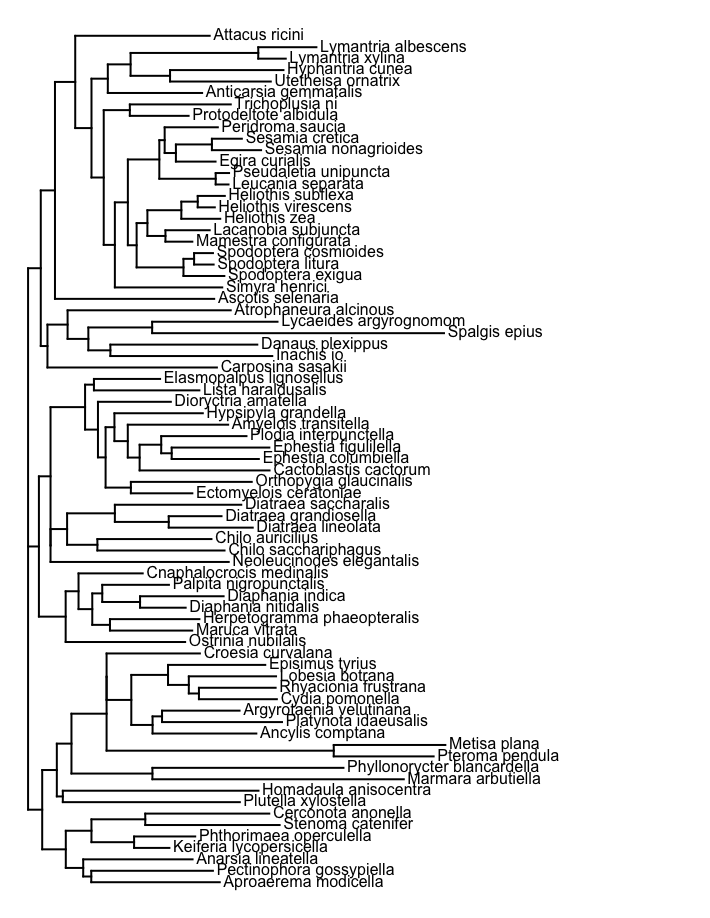


D_l_

| **Fixed effects** | **Estimate** | **SE** | **Z score** |
| --- | --- | --- | --- |
| absolute latitude | -0.087 | 0.0260 | -4.05 |
| egg | 23.5 | 0.896 | 30.04 |
| larva | 23.22 | 0.911 | 29.27 |
| pupa | 23.94 | 0.910 | 30.25 |
| **Random effects** | **SD** |  |  |
| species | 0.85 |  |  |
| residual | 1.18 |  |  |
| N = 167 obs., 71 species |  |  |  |

R^2^_lik_ (phylogenetic vs non-phylogenetic model): 0.0026

D_h_

| **Fixed effects** | **Estimate** | **SE** | **Z score** |
| --- | --- | --- | --- |
| absolute latitude | -0.048 | 0.029 | -1.623 |
| egg | 35.32 | 1.022 | 34.548 |
| larva | 34.97 | 1.010 | 34.651 |
| pupa | 34.91 | 1.011 | 34.514 |
| **Random effects** | **SD** |  |  |
| species | 0.004 |  |  |
| residual | 1.066 |  |  |
| N = 84 obs., 41 species |  |  |  |

R^2^_lik_ (phylogenetic vs non-phylogenetic model): < 0.0001

D_breadth_

| **Fixed effects** | **Estimate** | **SE** | **Z score** |
| --- | --- | --- | --- |
| absolute latitude | 0.031 | 0.025 | 1.276 |
| egg | 12.471 | 0.859 | 14.513 |
| larva | 12.012 | 0.835 | 14.386 |
| pupa | 11.088 | 0.843 | 13.139 |
| **Random effects** | **SD** |  |  |
| species | 0.001 |  |  |
| residual | 1.226 |  |  |
| N = 82 obs., 39 species |  |  |  |

R^2^_lik_ (phylogenetic vs non-phylogenetic model): < 0.0001

1. Phylogenetic tree used in T_0_ analysis


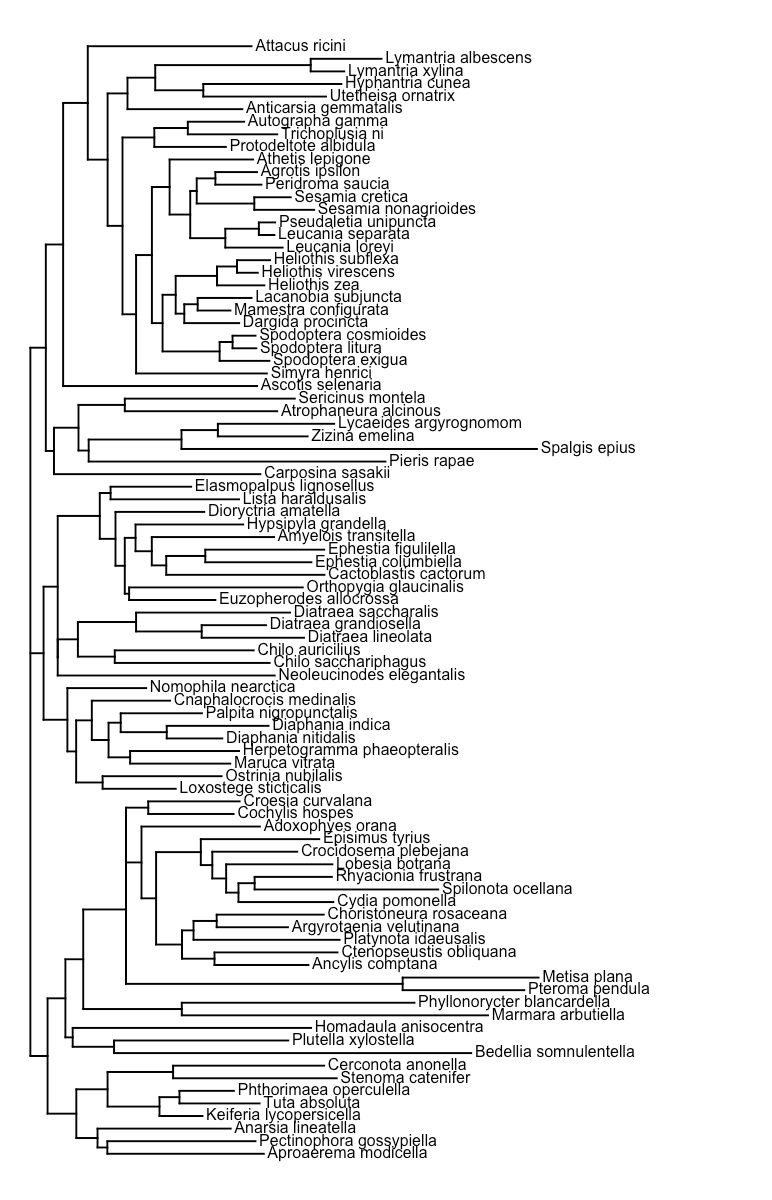


T_0_

| **Fixed effects** | **Estimate** | **SE** | **Z score** |
| --- | --- | --- | --- |
| absolute latitude | -0.0312 | 0.021 | -1.509 |
| egg | 11.43 | 0.835 | 13.700 |
| larva | 10.72 | 0.844 | 12.700 |
| pupa | 11.63 | 0.830 | 14.011 |
| **Random effects** | **SD** |  |  |
| species | 1.30 |  |  |
| residual | 1.583 |  |  |
| N = 235 obs., 89 species |  |  |  |

R^2^_lik_ (phylogenetic vs non-phylogenetic model): < 0.0001

1. Phylogenetic tree used in analysis of survival variables

**
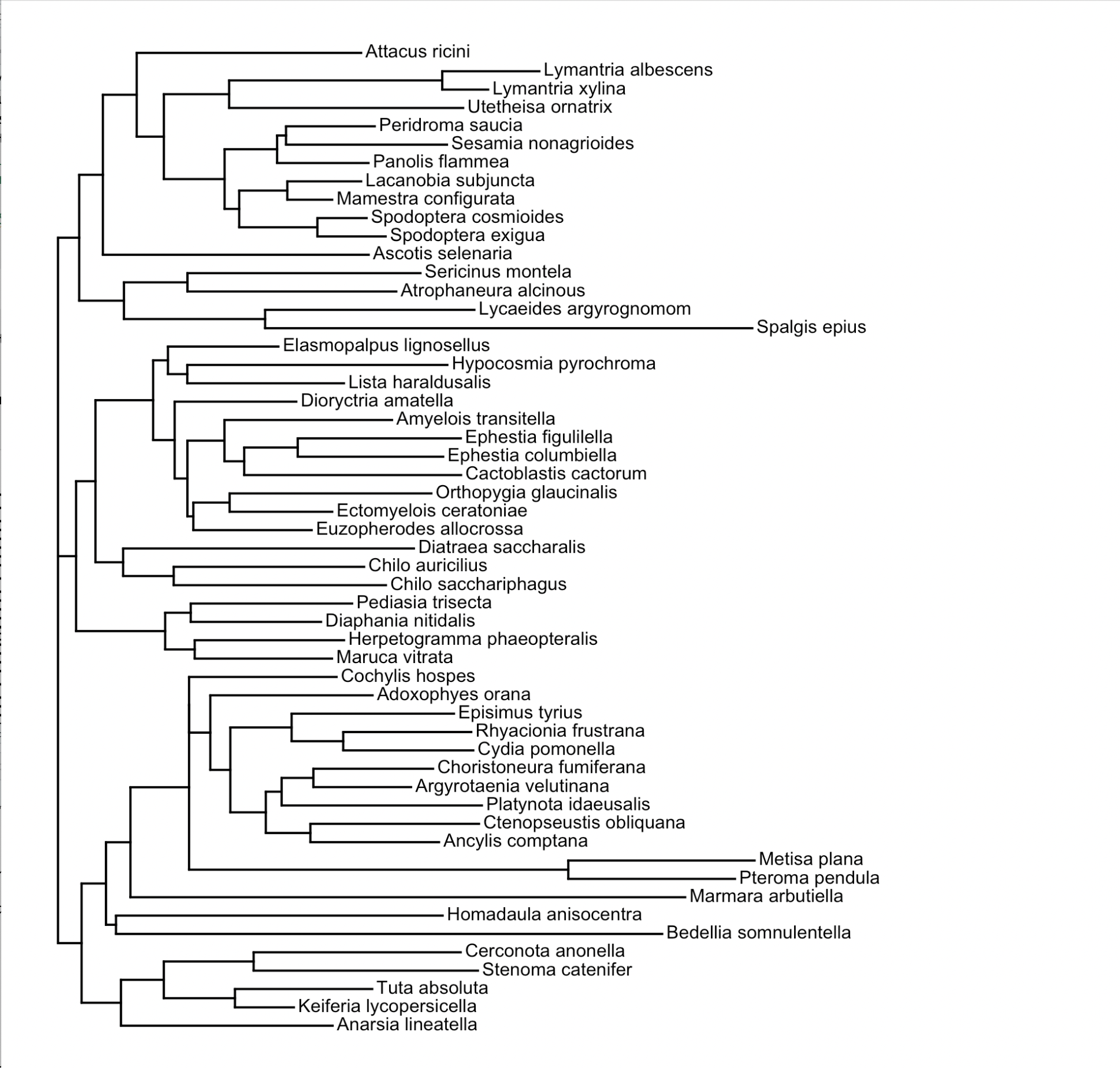
**

S_l_

| **Fixed effects** | **Estimate** | **SE** | **Z score** |
| --- | --- | --- | --- |
| absolute latitude | -0.258 | 0.053 | -4.837 |
| egg | 23.367 | 1.841 | 12.694 |
| larva | 20.569 | 1.692 | 12.161 |
| pupa | 19.108 | 2.044 | 9.346 |
| **Random effects** | **SD** |  |  |
| species | 1.92 |  |  |
| residual | 1.75 |  |  |
| N = 61 obs., 34 species |  |  |  |

R^2^_lik_ (phylogenetic vs non-phylogenetic model): - 0.02

S_h_

| **Fixed effects** | **Estimate** | **SE** | **Z score** |
| --- | --- | --- | --- |
| absolute latitude | -0.051 | 0.044 | -1.157 |
| egg | 33.710 | 1.603 | 21.026 |
| larva | 33.233 | 1.644 | 20.209 |
| pupa | 33.248 | 1.635 | 20.34 |
| **Random effects** | **SD** |  |  |
| species | 1.76 |  |  |
| residual | 1.75 |  |  |
| N = 65 obs., 36 species |  |  |  |

R^2^_lik_ (phylogenetic vs non-phylogenetic model): 0.003

S_breadth_

| **Fixed effects** | **Estimate** | **SE** | **Z score** |
| --- | --- | --- | --- |
| absolute latitude | 0.121 | 0.087 | 1.381 |
| egg | 13.333 | 3.199 | 4.167 |
| larva | 10.996 | 3.216 | 3.419 |
| pupa | 12.620 | 3.246 | 3.888 |
| **Random effects** | **SD** |  |  |
| species | 3.462 |  |  |
| residual | 2.415 |  |  |
| N = 41 obs., 24 species |  |  |  |

R^2^_lik_ (phylogenetic vs non-phylogenetic model): -0.287

1. Phylogenetic tree used in analysis of performance variables

**
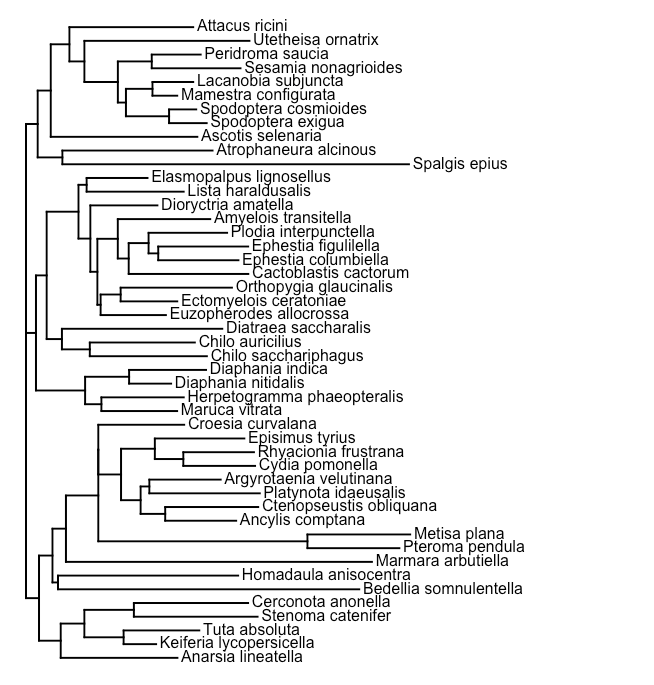
**

P_l_

| **Fixed effects** | **Estimate** | **SE** | **Z score** |
| --- | --- | --- | --- |
| absolute latitude | -0.109 | 0.036 | -3.069 |
| egg | 24.366 | 1.252 | 19.466 |
| larva | 24.424 | 1.285 | 19.012 |
| pupa | 24.881 | 1.271 | 19.574 |
| **Random effects** | **SD** |  |  |
| species | 1.355 |  |  |
| residual | 1.646 |  |  |
| N = 96 obs., 46 species |  |  |  |

R^2^_lik_ (phylogenetic vs non-phylogenetic model): -0.0263

P_h_

| **Fixed effects** | **Estimate** | **SE** | **Z score** |
| --- | --- | --- | --- |
| absolute latitude | -0.041 | 0.040 | -1.021 |
| egg | 33.979 | 1.383 | 24.56 |
| larva | 33.772 | 1.415 | 23.86 |
| pupa | 33.593 | 1.414 | 23.764 |
| **Random effects** | **SD** |  |  |
| species | 1.846 |  |  |
| residual | 1.073 |  |  |
| N = 62 obs., 36 species |  |  |  |

R^2^_lik_ (phylogenetic vs non-phylogenetic model): 0.023

P_breadth_

| **Fixed effects** | **Estimate** | **SE** | **Z score** |
| --- | --- | --- | --- |
| absolute latitude | 0.016 | 0.037 | 0.442 |
| egg | 11.177 | 1.261 | 8.860 |
| larva | 11.498 | 1.314 | 8.746 |
| pupa | 10.769 | 1.296 | 8.309 |
| **Random effects** | **SD** |  |  |
| species | 2.093 |  |  |
| residual | 1.589 |  |  |
| N = 60 obs., 34 species |  |  |  |

R^2^_lik_ (phylogenetic vs non-phylogenetic model): < 0.0001
