# Supplementary material for: How development and survival combine to determine the thermal sensitivity of insects
Source: PLoS One. 2024 Jan 30;19(1):e0291393. doi: 10.1371/journal.pone.0291393 (PMC10826953; doi:10.1371/journal.pone.0291393)
Supplement: Attachment — Submitted filename: ResponseToReviewers.docx [file pone.0291393.s005.docx]

Response to reviewers

We thank both reviewers for their positive feedback and constructive comments that allowed us to improve our manuscript. Please find below a list of the reviewers’ suggestions and descriptions on how we addressed each of them.

List of responses to reviewer comments:

Reviewer #1.

1- “I would like to offer a suggestion for improvement regarding the detailing of the construction of the phylogenetic tree. Please provide the reader with more details on the phylogenetic methods employed. This will provide more clarity on the phylogeny implications of your results. Once this concern is addressed, I am confident that your work will make a significant contribution.”

**We included the following statement in the revised manuscript explaining our phylogenetic methods (lines 311-318):**

**“To account for phylogenetic history, we custom built a phylogenetic tree for the 102 species used here. In short, we searched Genbank for 11 full-length, commonly sequenced genomic DNA markers, while accounting for taxonomic issues e.g. synonymy. Five species were not available on Genbank, and in those cases we utilized a congener as a surrogate. Sequences were aligned using mafft v7.294b [31]; Those locus alignments containing 10 or less species were removed, leaving a total of 8 usable loci. We used the resulting supermatrix and built an unpartitioned maximum likelihood tree using RaxML-NG v0.9.0 [32] under a GTR-G substitution model. See supporting information S3 for full details. With a phylogenetic hypothesis in hand, we fitted the models described above, but including the phylogenetic tree, using the R package *phyr* [33]”**

**We also updated Supplement 3, which describes in more depth the process outlined above, including a table of the DNA markers we used and a list of substitutions due to lack of DNA information.**

Reviewer #2:

Methods
1. I believe that this study would be reproducible from the methods as presented here. That said, I wonder if an infographic or flowchart of some kind would help the reader fully grasp the process described in lines 180-205? This is not vital for the publication of this manuscript, I think it would just add a little extra clarity if such a figure is possible to make.

**We sketched an infographic but did not consider that it would improve the clarity of the methods described in lines (now 196 to 217) so we decided to keep the original version of this section.**

2. Short title could be shortened and punchier.

**We changed the short title to “Insect thermal sensitivity”**

3. Line 85: Replace “lab” with “laboratory”

**We changed “lab” to “laboratory” as suggested (now line 116)**
4. Line 182: A comment has been left in the text to “insert data repository info”, which needs to be done

**We included Dryad information (line 223):- DOI: 10.5061/dryad.qjq2bvqk4**

5. Line 124: I would remove the semi-colon and split this sentence into two as follows “…full dataset. After comparison…”

**We think this refers to line 214 (now 235). We removed the semi-colon as suggested.**

6. Line 239-241: Would it not look better and be clearer if a formula was provided here? E.g. Performance = Standardized Development Rate x Standardized Survivorship. This will just help the reader find this easier if they are following the method and looking for the formula.

**We included the formula as suggested (lines 292-293):**

**“For each set, we standardized development rate (the reciprocal of development time) and survivorship so they varied between 0 and 1 (representing the maximum value) and subsequently multiplied them to obtain performance (Performance = Standardized Development Rate × Standardized Survivorship).”**

Results
7. Figure 3: Perhaps it is just the version of PDF that I downloaded, but the found the quality of all figures to be quite poor. This was particularly important for Figure 3, where I struggled to tell circles and squares apart as a result. I hope that the figures submitted for final publication are of a better quality.

**We updated all of our figures using PACE to make sure they meet PLOS format requirements.**

8. Table 1 and Figure 4: This table and figure are misplaced. They are first referred to in the paragraph starting on line 292, so I think they should be moved to line 306.

**We moved Table 1 and Figure 4 so they now appear at the end of the paragraph where they are first mentioned.**
